# Beef Essence

**Published:** 1897-04

**Authors:** 


### BEEF ESSENCE.

Dr. Joseph Adolphus says: "To make beef essence I take a cube of beefsteak, freed from fat, sear it over a charcoal or coke fire, or over an alcohol lamp that has a large flame; then I put it in a large lemon squeezer and squeeze out all the juice I can. This fluid contains five to ten per cent. albuminoids, a large quantity of the salts, and you may add a little new sweet milk and pepsin to it. I prepare the milk by curdling it with a few drops of dilute hydrochloric acid, then beat the curds with an egg-beater, add to it twenty or thirty grains of pure crystal pepsin. The milk thus prepared may be fed with the beef-juice; the two together make an admirable invalid food. I frequently rub the yolk of a hen's egg with half its bulk of glycerine, mix together; twenty to forty or sixty drops of this given every thirty to sixty minutes is a strong food, of quick absorption, and may be mixed with the milk prepared as above or with the beef essence.—*Ga. Ec. Med. Journal.*

